# Melanoma in people living with HIV: Immune landscape dynamics and the role of immuno- and antiviral therapies

**DOI:** 10.1007/s10555-024-10230-6

**Published:** 2024-11-29

**Authors:** Lindsay N. Barger, Olivia S. El Naggar, Binh Ha, Gabriele Romano

**Affiliations:** 1 Department of Pharmacology and Physiology, Drexel University College of Medicine, Philadelphia, PA USA; 2 Department of Microbiology and Immunology, Drexel University College of Medicine, Philadelphia, PA USA; 3 Immune Cell Regulation & Targeting Program, Sidney Kimmel Comprehensive Cancer Center Consortium, Philadelphia, PA USA

**Keywords:** Melanoma, HIV, Chronic infection, T cells, Immunotherapy, Cold tumors, Animal models

## Abstract

The intersection of HIV and melanoma presents a complex and unique challenge, marked by distinct patterns in incidence, mortality, and treatment response. Higher mortality rates among people with HIV who develop melanoma underscore an urgent need to identify the factors influencing these outcomes. Investigating immune system dynamics, the effects of anti-retroviral drugs, and the evolving landscape of cancer immunotherapy in this population holds promise for new insights, though significant uncertainties remain. Over the past 25 years, melanoma research has demonstrated that a robust immune response is critical for effective treatment. In the context of chronic HIV infection, viral reservoirs enable the virus to persist despite anti-retroviral therapy and foster dysregulated myeloid and T cell compartments. The resulting chronic inflammation weakens the immune system and damages tissues, potentially creating “cold” tumor microenvironments that are less responsive to therapy. In this challenging context, animal models become invaluable for uncovering underlying biological mechanisms. While these models do not fully replicate human HIV infection, they provide essential insights into critical questions and inform the development of tailored treatments for this patient population. Clinically, increasing trial participation and creating a centralized, accessible repository for HIV and cancer samples and data are vital. Achieving these goals requires institutions to address barriers to research participation among people with HIV, focusing on patient-centered initiatives that leverage biomedical research to improve their outcomes and extend their lives.

## Introduction

Anti-retroviral therapy (ART) has revolutionized the clinical management of people living with HIV (PLWH) by improving their length and quality of life. Among these improvements has been a significant reduction in the incidence of AIDS-defining cancers (e.g., Kaposi Sarcoma, cervical cancer). However, the incidence and mortality of non-AIDS-defining cancers (NADCs) have increased more than threefold in PLWH in recent years [1, 2]. Cutaneous melanoma is a malignant tumor arising from skin melanocytes, with ~ 100,000 new diagnoses and 7,000 deaths each year in the United States alone (American Cancer Society, 2023). PLWH have a slightly increased risk of developing melanoma but a substantially increased mortality rate (~ 4X) compared to the uninfected population with melanoma [3]. Furthermore, increased mortality occurs even in PLWH who are using ART, highlighting the growing need to develop an effective melanoma treatment that works alongside ART to target melanoma in PLWH. People with HIV can show variable disease progression according to HIV status, melanoma stage at diagnosis, or treatment disparities. Still, associations between HIV and melanoma-specific mortality remain significantly elevated even after adjusting for cancer stage or cancer treatment [3]. This suggests an underlying interaction between HIV infection and melanoma pathogenesis that drives melanoma progression. Nevertheless, the biological mechanisms underlying melanoma-specific mortality are heavily understudied in PLWH, who are still excluded from most oncology clinical trials. In this review, we discuss the clinical and pre-clinical evidence of the impact of HIV chronic infection on the immune system and the biological correlation with increased mortality rate for patients with melanoma. We point out critical gaps in the field and the possible future avenues to improve the research and clinical management of HIV and melanoma co-morbidity.

### Cutaneous melanoma: genetic alterations, incidence, and mortality

Cutaneous melanoma is an aggressive skin malignancy that develops in melanocytes, the pigment-producing cells in the skin. It is estimated that there were globally over 300,000 new melanoma cases and over 50,000 deaths in 2020, and this number is projected to increase [4]. Cutaneous melanoma is the most common and deadly among several melanoma subtypes, including mucosal and uveal [5, 6]. Risk factors for cutaneous melanoma include age and fair skin – many cases are attributed to exposure to ultraviolet radiation (UVR) [7]. Although cutaneous melanoma has a genetic component in some instances, it largely depends on the acquisition of somatic driver mutations. Such driver mutations contribute to disease initiation and progression. Frequently mutated genes include BRAF, CDKN2A, NRAS, NF1, PTEN, and TP53 [7, 8]. Over 50% of cutaneous melanomas harbor a hotspot mutation in BRAF, the most common being V600E [5]. Melanoma disease progression can include metastasis to the brain, lymph nodes, lungs, liver, and bone, among other places [9, 10]. Prognosis is generally poor for metastatic melanoma, and survival can be impacted by stage at diagnosis and metastatic site [11]. Brain-metastatic melanoma has an especially unfavorable prognosis. This outcome may be attributed to immune suppression in the tumor microenvironment, characterized by a decreased infiltrate of CD3^+^ T cells and monocytic lineage cells [12] and impaired drug delivery across the blood–brain barrier [13].

#### Response to therapy: the importance of an immunologically "hot" tumor microenvironment

The standard of care for melanoma patients is immune checkpoint inhibitor (ICI) therapy. Monoclonal ICI therapies like pembrolizumab and nivolumab target programmed cell death-1 (PD-1), while ipilimumab targets Cytotoxic T-Lymphocyte associated antigen 4 (CTLA-4) [14]. The clinical strategy can change depending on the resectability and staging of the tumors. For example, pembrolizumab was reported to show greater progression-free survival than ipilimumab [15], and combined anti-CTLA4 and anti-PD1 therapy shows increased overall survival compared to either single agent in unresectable, metastatic melanoma [16, 17]. Recently, anti-LAG-3 treatment has been approved for advanced melanoma in combination with anti-PD1 after showing excellent responses and a good toxicity profile [18]. For resectable tumors, neo-adjuvant anti-PD1 (i.e., administered before tumor surgical resection) appears particularly effective and will likely be a novel standard of care once optimal combinations and schedules are determined [19, 20]. As a second-line therapy for patients with BRAFV600E alterations, combined BRAF + MEK inhibitor treatment is generally utilized if immunotherapy fails. Tumor-infiltrating lymphocyte (TIL) therapy has recently been approved as a third-line option for treatment-refractory advanced melanoma patients if they are healthy enough to wait for T cell expansion and withstand possible side effects of the adoptive cell therapy process [21].

Despite increasingly effective tumor response and expanded therapeutic options, treatment resistance is still a major clinical issue. Resistance can be inherent (e.g., low expression of PD-1) or acquired, like decreased antigen presentation or alterations in interferon-receptor signaling pathways [22, 23]. In a pan-cancer study of survival following immunotherapy (that included melanoma), high tumor mutational burden (TMB) was shown to be associated with increased overall survival in patients receiving ICI. [24] High TMB can generate neoantigens, which may increase immunogenicity [25–28]. In general, patients with the most favorable long-term responses to targeted and ICI therapy display an elevated pro-inflammatory tumor immune infiltrate at baseline. This status is described as a "hot" tumor bed. Alternatively, those with lower pro-inflammatory infiltrate (a "cold" tumor bed) display less favorable outcomes to therapy [29]. Given this shortcoming, the field is evolving to develop strategies that will restore an immunologically active or "hot" tumor microenvironment. Multiple therapeutic approaches for melanoma are currently in pre-clinical and clinical trials. Some approaches aim to increase the recruitment of T cells and NK cells at the tumor site [30, 31], while others include the emphasis on novel checkpoint inhibitors like PSGL-1 or T cell immunoreceptor with Ig and ITIM domains (TIGIT) [32–34]. Novel strategies, such as fecal transplants, mRNA vaccines, and oncolytic viruses, are also under investigation [35, 36].

Aside from increasing T cell homing and activation, targeting immune suppressive populations, like myeloid-derived suppressor cells (MDSCs), may be a novel avenue to modulate the immune system in the tumor microenvironment [37]. MDSCs are a category of cells that inhibit pro-inflammatory signaling – both the innate and adaptive immune response. Patients who bear elevated levels of MDSCs show poor prognosis in cancer treatment, and they have been associated with a lack of melanoma-specific T cell responses [38, 39].

Overall, a lesson learned in the last 25 years of pre-clinical and clinical research in melanoma is that there is a strong relationship between response to therapy and an effective immune response against the tumor in all therapeutic approaches available. In the following sections, we will introduce the primary immunologic mechanism by which chronic HIV infection alters the baseline immune response, potentially contributing to the formation and maintenance of "cold" tumor beds.

### Long-term effects of HIV-1 chronic infection on the immune system

**Fig. 1:**
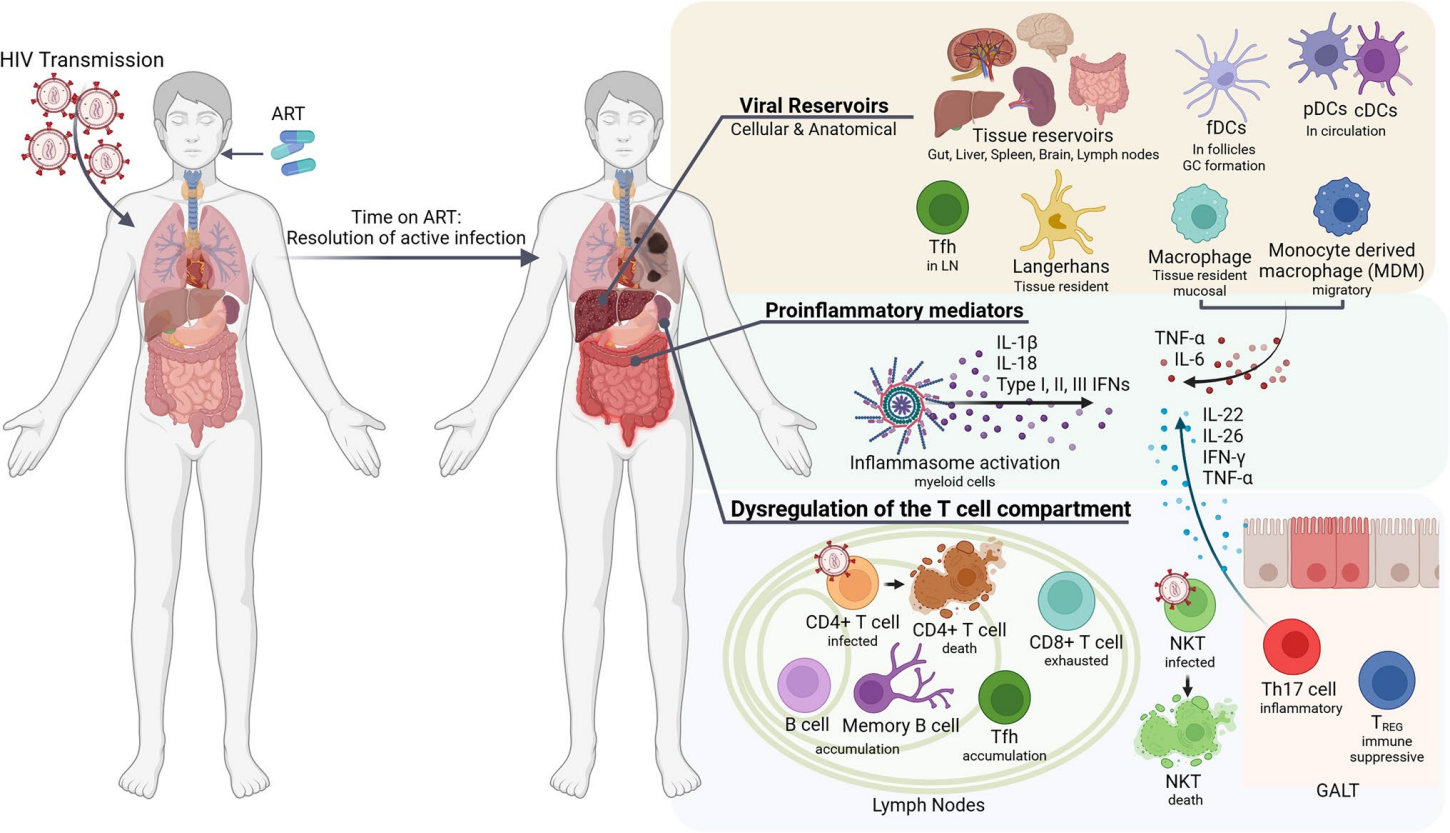
HIV chronic infection is fostered by the viral reservoir, causing dysregulation within the T cell compartment, and sustained expression of pro-inflammatory mediators. Long-term effects of HIV infection occur also in patients with controlled infection and on ART. fDCs, follicular dendritic cells; pDCs, plasmacytoid dendritic cells; cDCs, conventional dendritic cells; T_FH_, follicular helper T cells; ART, anti-retroviral therapy; GALT, gastrointestinal-associated lymphoid tissues; T_REG_, regulatory T cells

In the ART era, HIV-1 infections have become manageable but, unfortunately, remain a chronic disease. HIV-1 transmission and infection are followed by substantial damage to lymphoid tissues and immune cell microenvironments [40]. The immune response to HIV-1 infection perpetuates this tissue damage and has lasting effects on immune competency after ART initiation [40]. HIV-1 spreads through mucosal barriers during unprotected sexual contact, childbirth, or needle sharing. Following transmission, the virus integrates into the genome, establishing latent reservoirs. Peak viremia stages follow, with viral RNA detectable as immune responses begin [40]. After disseminating to secondary lymphoid organs and peripheral tissues, a systemic inflammatory response ensues, leading to a viral set point maintained until significant CD4 + T cell loss results in acquired immunodeficiency syndrome (AIDS). Theoretically, very early application of ART can prevent integration, prevent CD4 + T cell death, and rescue HIV-1 specific T cell responses. However, early ART is challenging to implement, as viral integration and latent reservoirs are established within 5–10 days of transmission, which is well before viremia is clinically detectable in plasma and before ART can be initiated [40, 41]. In spite of successful ART regimens that block the virus from spreading to new cells and drive undetectable viremia, HIV, once integrated, remains a permanent infection and contributes to persisting immunological changes that impact the ability to fight off infections and cancers, among others [40]. Commonly, PLWH on ART suffer from low-grade chronic inflammation, even after HIV-1 becomes undetectable. The three primary factors contributing to this chronic inflammation are: the viral reservoir, the dysregulation within the T cellcompartment, and the sustained release of pro-inflammatory mediators from activated myeloid cells **(**Fig. 1**)**.

#### Establishment and maintenance of the viral reservoir

In the context of chronic inflammation in HIV, innate and adaptive immune cells play a crucial role in establishing and maintaining the pathogenic effects of viral infection. For the transport and dissemination of the virus, innate immunity is the main contributor. Langerhans cells, a subset of dendritic cells (DCs), are among the first cells to encounter HIV-1 during transmission, facilitating the virus’s passage through mucosal barriers [42]. By similar mechanisms, migratory conventional DCs (cDCs) contribute to transferring the virus to CD4 + T cells and to secondary lymphoid organs [43], while follicular DCs (fDCs) maintain germinal centers and trap HIV, enhancing susceptibility to infection [44].

Macrophages, present in most tissues, are also major reservoirs of HIV-1 infections that persist during ART [45]. Tissue-resident macrophages do not migrate to lymph nodes, unlike DCs, but instead, present HIV-1 antigens to CD4 + T cells in the mucosa [45]. Resident macrophages can be infected with HIV-1 and then transmit the virus to CD4 + T cells via cell-to-cell contact [46]. During the acute phase of HIV infection, monocytes recruited to the site of infection differentiate into macrophages. Here, they can be infected with HIV-1 and migrate from the site of infection to almost any tissue of the body. HIV-1-infected macrophages are central in seeding HIV-1 infections in gastrointestinal tissues, the brain, liver, lung, and more [47].

#### Dysregulation of the T cell compartment

HIV-1 significantly impacts the T cell compartment, leading to AIDS through persistent CD4 + T cell loss. ART allows for the restoration of peripheral CD4 + T cell populations, but memory CD4 + T cells are often depleted early in infection [48]. The pool of memory CD4 + T cells can be regenerated (even if not entirely) in blood and secondary lymphoid tissues after ART initiation [49, 50]. Nevertheless, HIV-1-infected memory T cells have shortened telomere lengths that contribute to premature senescence, permanent cell exhaustion, increased T cell turnover, and premature aging of the entire immune system [51, 52].

HIV-1 preferentially infects follicular helper T cells (T_FH_) due to their expression of CCR5. High levels of viremia and untreated HIV-1 infections result in the accumulation of T_FH_ cells in the lymph nodes [53, 54], followed by an accumulation of proliferating B cells in the germinal centers [53]. Specific neutralizing antibodies will rarely be generated against the envelope proteins due to their rapid mutation rates and different glycosylation states [55]. There is evidence that HIV-1 can cause B cell hyperactivity, resulting in hypergammaglobulinemia, increased turnover of B cells, autoantibody production, B-cell malignancies, and increased upregulation of CD80 and CD86 [56]. Memory B cells are often incompletely restored even after ART [56].

Th17 cells are a unique subset of CD4 + T cells that produce the highly inflammatory cytokine IL-17 [57, 58]. HIV-1 infection of Th17 results in cell death, which can raise the risk for bacterial or fungal co-infections, and cell death itself increases the systemic inflammatory response in the gastrointestinal-associated lymphoid tissues (GALT) [59]. The levels of Th17 cells can be reconstituted in some patients with ART [60].

Regulatory T cells (T_REGs_) play dueling roles in infection. They suppress the activation of inappropriate immune responses, thereby limiting tissue inflammatory damage. Total T_REG_ frequencies are increased during HIV-1 infection but can be normalized with ART [61, 62]. However, T_REGs_ in this environment have a higher level of activation and expression of immunosuppressive and migratory markers, which are not affected by ART [62]. Therefore, even on ART, T_REGs_ contribute to immune dysfunction, GALT fibrosis, and chronic inflammation over time [62].

HIV-1 stimulates CD8 + T cell response, which can initially control the virus in acute infection, but cytotoxicity dwindles throughout the infection [63]. CD8 + T cells in uncontrolled and late infections have a reduction in naïve levels, increased activation and exhaustion markers, and impaired cytokine and perforin/granzyme production [64]. Notably, ART does not fully reconstitute the CD8 + T cellcompartment functionality.

Natural Killer T cells (NKTs) are a special type of lymphoid innate immune cells. They are important early immunoregulatory cells and aid in anti-tumor immune responses [65]. NKT cells are selectively depleted from circulation over the course of HIV-1 infections [66, 67]. These cells are affected more by R5 tropic viruses than X4 [67]. NKT cells may be restored with the initiation of ART, but CD4^−^ NKT subpopulations return more robustly than CD4^+^ NKT subpopulations, and overall NKT numbers are lower than physiological infection-free levels [66, 67].

#### HIV-induced chronic inflammatory status

After viral reservoir seeding and HIV-1 dissemination to lymphoid organs, HIV-1 infections are clinically detectable, and ART can be initiated. However, a baseline chronic inflammatory status has been established by this time, fostered by cells and cytokines of the immune system, especially of the myeloid compartment. One source of robust inflammation is the recognition of pathogen-associated molecular patterns (PAMPs) by their respective pathogen recognition receptor (PRR) to initiate the formation of the inflammasome. The classical NOD-like receptor family pyrin domain containing 3 (NLRP3) inflammasome in monocytes and macrophages is activated by TLR8 sensing of HIV-1, triggering the production, processing, and release of IL-1β and IL-18 [68]. IL-1β, essential for innate immunity, increases susceptibility to HIV-1, exacerbates inflammatory tissue damage, and promotes bacterial translocation from the intestinal lumen into circulation [69, 70]. In addition to NLRP3, many other inflammasomes contribute to chronic inflammation, like CARD8 and AIM2.

In theory, ART should decrease HIV replication and, therefore, decrease inflammasome activation in response to HIV PAMPs [71]. However, data that supports this theory in inflammasomes other than NLRP3 is extremely limited. Notably, inflammasomes can be activated in response to not only viral PAMPs but bacterial PAMPs as well. Pathological effects of HIV infection can damage gastrointestinal mucosal barriers to permit passage of bacteria or bacterial products from the gastrointestinal tract into the blood, termed microbial translocation (MT). MT is incompletely restored with ART [72], contributing to persistent inflammasome activation, tissue damage, and the chronic inflammatory pathology seen in PLWH on ART [73].

Persistent production of pro-inflammatory mediators, like TNF-α, may induce the release of suppressive factors such as CCL5 [74, 75]. The chemokine CCL5 is expressed early in HIV infection as it is a natural inhibitor for HIV-1 infections, directly competing for the use of the co-receptor CCR5 [76]. Abundant CCL5 has been associated with many inflammatory disorders, cancers, and HIV-associated neurocognitive disorder (HAND). Elevated CCL5 is also associated with the recruitment of MDSCs, a heterogeneous population of immature myeloid cells that suppress immune responses, particularly T cell responses. As immunoregulatory cells, MDSCs mediate the anti-inflammatory immune response and support immune tolerance [77]. As pathological cells, MDSC accumulation is associated with poor prognoses in states of infection or disease [77, 78]. In the case of HIV-1 infections, MDSCs are theoretically expanded to tamper the initial immune response and chronic immune activation seen in HIV-1 infection, but their accumulation may enable viral persistence and evasion of immune detection [77]. Persistent MDSCs are found in PLWH during chronic stages of an active HIV infection, as well as PLWH on ART [79]. In these patients, MDSCs mediate the expansion of T_REGs_, induce an exhaustive phenotype in CD8 + T cells, and introduce an immunosuppressive environment [77, 79]. MDSCs have the capacity to be infected with HIV-1, expressing CD4, CCR5, and CXCR4. Still, infection is not likely deleterious to these cells as they increase as the infection progresses. However, their pathological function may change depending on the local environment [79].

The chronic immune activation and inflammation seen in PLWH on ART is not only linked to dysregulated immune responses but also to the profound fibrosis of lymphoid tissues (LT), particularly the GALT, through collagen deposition and damage to the fibroblastic reticular network (FRC) [80]. Importantly, ART does not reverse collagen deposition or restore CD4 + T cell sub-populations in these tissues, even after years of ART or in patients considered elite controllers [81, 82].

Overall, the chronic impact of HIV-1 infection extends far beyond the mere presence of the virus itself, leaving an enduring legacy within the immune system. The intricate interplay between viral persistence, dysregulated immune cell functions, and sustained inflammatory mediators orchestrates a landscape conducive to immunological perturbations. The establishment and maintenance of viral reservoirs through diverse immune cell subsets, particularly innate immune cells, paves the way for continual viral dissemination and persistence even under anti-retroviral therapy (ART). Concurrently, the dysregulation within the T cell compartment, marked by the depletion of various CD4 + T cell subsets and alterations in their functionalities, amplifies the immune dysfunction. Moreover, the sustained chronic inflammatory status, characterized by the activation of multiple inflammasomes, perpetuates immune activation and tissue damage. Hence, the enduring repercussions of HIV-induced chronic inflammation create an environment primed for an impaired immune response, fostering favorable conditions for the formation of an immunologically "cold" tumor microenvironment. In the following sections, we analyze the biological evidence that connects HIV-induced immune dysregulation and clinical observations in melanoma patients, focusing on the critical challenges in the field and especially on the observed increased melanoma-specific mortality in PLWH.

### HIV and melanoma co-morbidity

The co-occurrence of HIV and melanoma sparks complex questions about the relationship between incidence rates, mortality risks, immune dynamics, and therapeutic implications. In this section, we analyze the literature about the many factors involved in this group of patients, ranging from augmented medical screenings, to UV exposure variations, to demographic peculiarities.

#### Melanoma incidence in PLWH

While many NADCs have shown a substantial increase in incidence in the post-ART era (e.g., liver cancer), the incidence of melanoma appears to be only slightly increased in PLWH [83–86]. Several studies have detailed the epidemiology of HIV and melanoma co-morbidity [87]. A systematic review on cancer incidence in PLWH has recently collected 25 published studies (1165 cases total) evaluating Standardized Incidence Rate (SIR) for various NADCs, including melanoma, and found that, overall, PLWH do not seem to have a significantly higher SIR than the uninfected population (SIR 1.19), but the body of data shows high variability [2]. For example, a study in the San Francisco area showed significantly increased SIR in MSM (Men who have Sex with Men) with AIDS [88]. While this might be due to decreased immune surveillance, the study posited that this increase was probably caused by a mix of factors in this category of patients: a combination of increased medical screening (people with AIDS get screened frequently for Kaposi Sarcoma lesions), and exposure to ultraviolet UV (increased probability of using tanning beds) [88, 89]. This last hypothesis seems to be supported by other studies with mostly white male patients with higher exposure to UV light (e.g., Mediterranean areas [90]) but unconfirmed in different populations. For instance, a study on Taiwanese patients indicated a significantly increased SIR in melanoma among HIV-infected individuals (SIR 7.96) [91], but the incidence of melanoma in the Taiwanese population is not associated with UV exposure [92]. Remarkably, when an increase in melanoma is detected in specific subgroups of patients, it does not appear to correlate with a decline in CD4 + T cell count at diagnosis or general immune suppression, and SIR does not increase over time after diagnosis [88, 93]. Sporadic outlier studies, such as one performed in southeast England, found a significantly decreased risk of cutaneous malignant melanoma among HIV-infected men (SIR 0.2) [94], possibly due to under-ascertained or misclassified Kaposi Sarcoma. In conclusion, while a number of studies have observed analogous melanoma risks between uninfected and infected individuals, noteworthy exceptions exist. When an increased melanoma incidence is observed in specific subgroups, it is not necessarily linked to a decline in CD4 + T cell counts or overall immune suppression. There is a dire need for better organized and detailed population studies on this topic to understand how environmental factors (e.g., UV exposure), demographics (e.g., race and ethnicity), immune status, and ART affect the risk of developing melanoma in PLWH.

#### Melanoma mortality in PLWH

The mortality rate among PLWH who develop melanoma is significantly elevated, approximately 2 to 4 times higher than that of the uninfected population with melanoma (average Standardized Mortality Rate (SMR) 3.95) [2]. Considerable variability is observed in the handful of studies that analyze this aspect [3, 95]. For example, an Italian study [95] indicated an SMR greater than 10 for PLWH with melanoma. Even if reports on melanoma-specific mortality in PLWH are scarce, it is clear that PLWH fare much worse than the uninfected population with melanoma. For example, PLWH are more likely to be diagnosed with distant-stage melanoma [96, 97].

The first components to consider are psycho-social factors common for all PLWH with malignancies. For instance, PLWH may delay seeking healthcare due to the fear of HIV-related stigma from healthcare providers, leading to more advanced cancer stages at diagnosis [96, 98]. Notably, in the US, PLWH are less likely to have health insurance and less likely to receive cancer treatment [96, 99]. Moreover, PLWH are less likely to be treated in Comprehensive Cancer Centers, where multidisciplinary teams are more likely to work together (dermatologists, HIV specialists, and oncologists) [100]. Oncologists perceive PLWH as prone to treatment-related side effects, leading to fewer standard treatment recommendations [100, 101]. Stage I-III patients are less likely to receive treatment and more likely to receive palliative care, whereas Stage IV PLWH receive less palliative care assistance than the uninfected counterpart. This highlights a significant problem not only in cancer treatment outcome but also in the quality of life management of this class of patients [100] in terms of managing symptoms, providing emotional and psychological support, enhancing communication and decision-making, and promoting a holistic approach to care that considers all aspects of the patient’s well-being.

Nevertheless, the association between HIV and melanoma-specific mortality remains significant even after adjusting for cancer stage or healthcare factors [3, 96]. This scenario could be attributed to multiple factors. Indeed, the biology underlying melanoma and HIV is strongly understudied, as PLWH have been and still are excluded from most oncology trials. Interestingly, as observed for incidence, there seems to be no correlation between CD4 + T cell count and overall survival [102], indicating that increased melanoma mortality in PLWH might not be related to general immune suppression but to more finely regulated factors. Nevertheless, an overall better immunological status is desirable for any cancer patient, and patients with higher CD4 + T cell counts had a significantly prolonged time to melanoma relapse and would be less likely to develop further complications such as opportunistic infections [102].

The increased mortality risk persists even in PLWH who are on ART regimens. Still, HIV-positive patients who are not treated for HIV or receive only single-agent ART show a worse prognosis than the ones on combination ART regimens [102]. Another essential aspect to consider is the number of tumor-infiltrating lymphocytes (TIL), as low TIL levels have been repeatedly reported in PLWH with melanoma [87, 103, 104]. If future studies confirm that PLWH have more probability of non-brisk TIL type, this would likely be an actionable therapeutic area of interest. While ICI does not perform well in cold tumors, many other approaches have been proposed to turn ’cold’ tumors into ’hot’ ones [29, 105].

No specific guidelines for the clinical management of PLWH with melanoma have been established [106–109]. Chronic exposure to HIV-1 antigens triggers follicular hyperplasia and lymphadenopathy early in infection. As the disease progresses, follicular health declines, germinal centers diminish, and fibrosis increases in lymph nodes [110]. Hence, the choice between surgical and ultrasonography options ideally necessitates a case-by-case discussion, but the latter approach might pose a greater risk of missing a diagnosis in PLWH [87]. Nevertheless, even if lymph nodes are analyzed, evaluating metastasis biomarkers like S-100β and HMB-45 might be problematic, as many of these markers have been reported to be impacted by HIV infection [87, 111–113]. In conclusion, the factors influencing the increased melanoma mortality rate in PLWH are multifaceted, including delayed healthcare seeking, immune suppression, and potential drug interactions from treatments (see below). More research is needed to understand melanoma-specific mortality in PLWH and develop tailored diagnostic and therapeutic strategies.

#### The effects of ART therapy on the immune dynamics in PLWH

The effects of ART on immune surveillance in PLWH are heavily understudied. A major barrier to studying ART is that a typical ART regimen is a combination of three HIV medications from two or more drug classes. This regimen is often adjusted multiple times during a patient’s lifetime. As a result, drawing definitive conclusions is challenging; however, it cannot be overlooked that these potent drugs are administered chronically and may impact immune dynamics. For example, ART may lead to a significant reduction in immune surveillance in the skin by decreasing the number of resident T cells [114]. The decrease in T cell surveillance appears to be prevalent in PLWH who receive ART "late" after infection (average 50 months), which, according to the CDC, is close to the median time PLWH realize they have been infected (3–5 years). Late ART patient samples showed a reduction of resident Th1 cells, dysregulation of the CD8 + T cell compartment, and a tolerogenic skin immune microenvironment with increased percentages of Th2 cells. This phenomenon may contribute to creating an immune suppressive/evasive microenvironment within the skin of PLWH, potentially making them more susceptible to "cold" tumors that are less responsive to conventional cancer treatments [29].

In addition, ART drugs have potential anti-cancer effects on cancer cells. Nelfinavir, an older protease inhibitor, has shown anti-tumor activity by inhibiting melanoma cell proliferation and suppressing pathways linked to metastasis and resistance to MAPK inhibitors [87, 115]. Maraviroc, another anti-retroviral drug that antagonizes CCR5, improved immune responses in melanoma by increasing CD4 + and CD8 + T cell infiltration and remodeling the tumor microenvironment [116]. Even though some of these ART drugs are not widely used (e.g., maraviroc) because better strategies for HIV management have been developed, it might be worth re-evaluating them in the context of cancer co-morbidity as a tailored regimen for PLWH.

#### Cancer immunotherapy in PLWH

Cancer immunotherapy in PLWH has been a vastly understudied topic as PLWH have been, and still are, readily excluded from clinical trials [117]. PLWH were considered ineligible for clinical trial participation because of possible interference with ART drugs, immune suppression, or occurrence of immune-related adverse events (irAE). It is estimated that more than 70% of PLWH have been excluded from melanoma immune checkpoint inhibitors (ICI) clinical trials, even after the FDA 2020 revised guidelines on ICI in PLWH [117]. In the last few years, research has shed some light on the relationship between immune checkpoints and HIV persistence. For instance, in PLWH, CD4 + T cells expressing PD-1, LAG-3, and/or T cell immunoglobulin and ITIM domain (TIGIT) have been found to harbor HIV DNA at a higher frequency, particularly in cells co-expressing multiple immune checkpoints [118–121]. As a result, increased immune checkpoint expression can promote viral latency and impair the cytotoxic function of HIV-specific T cells. This suggests that immune checkpoint inhibitors might yield two distinct effects in PLWH receiving anti-retroviral therapy (ART): 1) ICI could potentially activate HIV expression in latently infected CD4 + T cells, making them susceptible to immune recognition and/or viral-induced apoptosis (i.e., ’shock and kill’); 2) they can reinvigorate exhausted T cells and enhance HIV-specific T cell function [118].

Studies on non-oncologic PLWH show that anti-PD-L1 and anti-PD-1 elicited mixed results, with some individuals experiencing increased HIV-specific CD8 + T cell responses but no significant impact on plasma or cell-associated HIV [122].

The study of the immunological mechanisms in the intricate network of melanoma, HIV, and immune checkpoints is limited to a few case studies; hence, the conclusions that can be drawn are rather limited. It appears that, in terms of HIV parameters (e.g., viral titer, CD4 + T cell counts), immunotherapy has generally not shown a deleterious impact in melanoma patients [123, 124]. Some patients experienced positive treatment responses without compromising immune function or apparent interference with ART therapy [125–131]. There is an episodic report of irAE, but most of the reactions were Grade 1–2, not requiring treatment discontinuation, in line with data in the uninfected patients [123, 126, 129, 132]. Even patients with baseline CD4 + T cell counts < 200 cells/mL, VL ≥ 400, or opportunistic infections tolerate ICI well and do not show increased irAE compared to the uninfected population [123]. Also, when patients did not experience anti-tumor benefits from therapy, the HIV parameters (e.g., viral titer, CD4 + T cell counts) were unchanged or improved during treatment [126, 133]. Some studies reported transient changes in viral load, showing the occurrence of the "shock and kill" phenomenon [123, 127]. For instance, the combination treatment with anti-CTLA4 and anti-PD1 can reverse HIV latency in some patients and enhance HIV-specific T cell function. Central and effector memory responses to Gag stimulation were increased, as well as augmented effector memory CD4 + T cells (overall increase in IFN-γ and TNF-α production in response to stimulation). While the effect on HIV-specific T cells seems to be confirmed by multiple studies, the impact on HIV viral reservoir is only sporadic and mostly transient, and mainly in anti-CTLA4-treated patients, indicating that ’shock and kill’ might be happening only in specific patient subsets [123, 125, 130, 134].

We are currently in the dawning era of immune checkpoint inhibitor treatment for PLWH with cancer, and a significant portion of the underlying biology is presently unclear. While it is already evident that ICI is not less safe for PLWH than in the uninfected population [135–137], the overall effects on immunological dynamics are rather obscure. Specifically designed clinical studies must address how ICI alters HIV parameters (e.g., biomarkers for viral latency reversal) and how chronic HIV and its treatment affect the response to ICI in oncologic patients. For melanoma, there is the need to assess which are the best ICI combinations and how feasible/advantageous the neo-adjuvant vs. adjuvant setting in PLWH with resectable tumors is. Also, the technical and clinical feasibility of TIL therapy is entirely to be investigated in PLWH, as this treatment approach has only been recently approved.

**Fig. 2:**
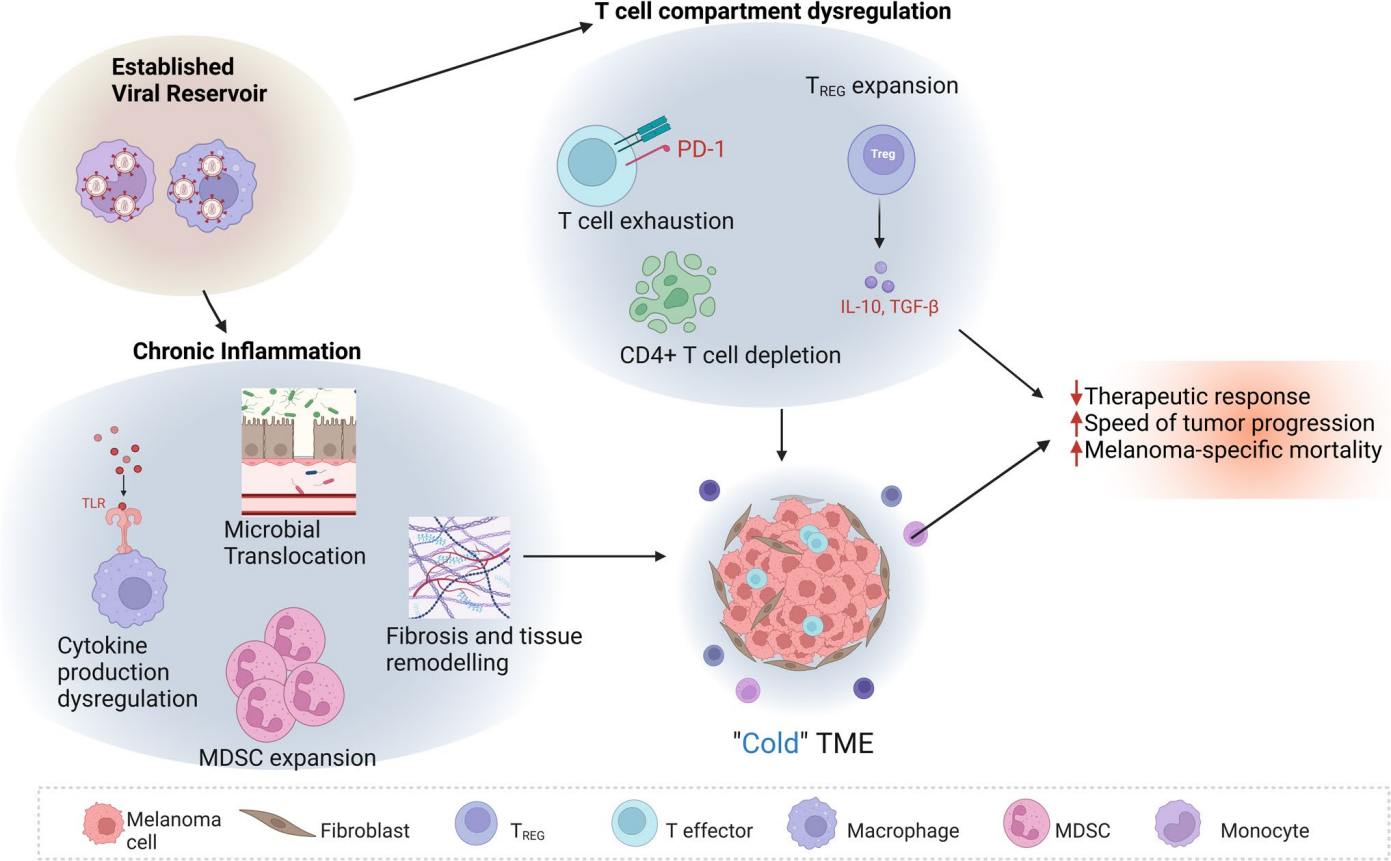
The three primary factors contributing to HIV chronic inflammation (the viral reservoir, the dysregulation within the T cell compartment, and sustained expression of pro-inflammatory mediators) can be strong contributors to the formation of a cold tumor microenvironment (TME), eventually causing decreased response to therapy and increased melanoma mortality. MDSC, myeloid-derived suppressor cells; TLR, Toll-like receptor

The convergence of HIV and melanoma portrays a complex interplay marked by nuanced trends in incidence, mortality, immune dynamics, and therapeutic responses (Fig. 2). The amplified mortality rates among PLWH diagnosed with melanoma highlight the need for a deeper understanding of the multifaceted factors influencing the outcomes. Integrating clinical research with systematic studies of the microenvironment of HIV + patients could lead us to identify novel markers of treatment response, diagnosis, and prognosis and eventually shape the best standard of care for PLWH with melanoma.

### Proposed experimental models of HIV and melanoma co-morbidity

As of today, no models of HIV and melanoma co-morbidity have been reported. Modeling HIV and cancer as a co-morbidity presents numerous challenges due to the complex interactions between cancer cells, chronic HIV infection, and the immune system. Cell culture and co-culture systems might be a strategy for answering specific questions, such as the interaction of HIV + immune cell subtypes with cancer cells or the molecular details of a signaling pathway, but they lack the comprehensiveness and depth of a living system. From an experimental point of view, a major complication is given by the fact that HIV-1 does not infect rodents [138], requiring a modification of the virus and/or of the animal models to reproduce the human illness. Other models use viruses that are like HIV but are species-specific (e.g., SIV) and require large animal species, with cost and ethical considerations attached. Even if no models of HIV and melanoma co-morbidity have been developed as of today, several strategies have the potential to be integrated and complemented and inform the field on biomarkers, therapeutic approaches, and diagnostic tools.

#### Animal models of HIV and cancer

Non-human primates remain the widely used model to study HIV/AIDS disease pathogenesis, but their use in cancer research is limited by ethical concerns, costs, and scalability [139]. Rodents have become the elite models for oncology studies, but they have the basic issue of un-infectability with HIV-1. A possible approach is to sidestep HIV-1’s infectious phase by integrating its genome directly into a rodent model, creating a non-infectious model expressing HIV-1 genes in multiple tissues without requiring high-level biosafety labs [140–142]. These models do not mimic initial HIV infection or the stages of AIDS progression but shed light on how accumulated viral gene products drive HIV-related disease progression (Table 1). In Tg26 mice, integrating the proviral plasmid with specific gene deletions results in transgenic animals manifesting AIDS-like symptoms in the lymphoid tissues, the kidneys, and the skin, among others [142–144]. While this is an excellent model to study the effect of viral proteins on the immune system, it lacks most of the aspects of HIV as a chronic infectious disease that are fundamental to cancer co-morbidity, and these animals have a relatively short-lived lifespan (< 1 month homozygous, 2–6 months heterozygous). A similar rat model has also been established but retains all the issues of the mouse counterpart [140, 141, 145]. Another interesting transgenic model utilizes the CD4 human promoter to express the full sequence of HIV-1 (CD4C/HIV). These mice develop AIDS-like symptoms with significant impairment of the T cell compartment but retain most of the problems of the Tg26 system and show even shorter lifespans [146, 147]. Various versions of these models have been developed, differentially expressing HIV-1 genes (e.g., Nef, Vpr), and they are valuable systems to detail the impact of single or multiple viral proteins on specific immunologic and physiological processes.

**Table 1:** Transgenic and transplant rodent models of HIV-1 infection

Model	Strategy	Advantages	Disadvantages	References
HIV-1Tg rats	Transgenic expression of HIV-1 provirus with a functional deletion of gag and pol	- Shows expression of viral proteins in most tissues, including lymphoid tissues and the skin - Several chronic HIV infection effects on lymphoid tissues are observed, such as selective loss of splenocytes, increased apoptosis of endothelial cells and splenocytes, follicular hyperplasia of the spleen - Good model to study the effect of viral proteins on the immune system and neurocognitive impairment - Relatively long lifespan (> 20 months) - Rats develop a kidney disease identical to human immunodeficiency virus (HIV)-associated nephropathy (HIVAN) observed in HIV-infected patients of African descent	- Non-infectious model - Rodent tissues and physiology - No development of CD4 + T- cell depletion or AIDS-like symptoms	[145, 148]
Tg26 mice	Transgenic expression of HIV-1 provirus (10–20 copies) with a functional deletion of gag and pol	- mRNA expression of HIV in skin, skeletal muscle, and kidneys - Mice develop HIVAN observed in HIV-infected patients of African descent - Heterozygous mice exhibit several AIDS-like symptoms like severe ventral subcutaneous edema, reduction in the size of the thymus and lymph nodes, and skin lesions - Several versions exist with differentially expressed HIV-1 proteins	- Non-infectious model - Murine tissues and physiology - In heterozygous animals, premature death is observed (between 2 and 6 months) - Heterozygous mice do not develop CD4^+^T cell depletion -Homozygous mice rarely survive to weaning	[143, 144]
CD4C/HIV mice	Transgenic expression of HIV-1 virus under the human CD4 gene promoter sequences	- Development of severe AIDS-like disease with a low percentage of mature CD4^+^ and CD8^+^ T cells - Development of muscle wasting, severe atrophy and fibrosis of lymphoid organs, tubulointerstitial nephritis, and lymphoid interstitial pneumonitis - Increased expression of Ccl5 in various tissues of these Tg mice compared to controls -Several versions exist with differentially expressed HIV-1 proteins	- Non-infectious model - Murine tissues and physiology - Very short life span (< 1 month)	[146, 147, 149]
hCD4/hCCR5 Tg mice	Transgenic expression of human CD4 and CCR5	- Infectable with HIV-1 R5-tropic strains using traditional routes of infection (e.g., mucosal) - Usable for preventative studies (e.g., vaccines, neutralizing antibodies) - Myeloid cells show elevated HIV-1 infection if Tg codes also for Cyclin T1	- Decreased HIV-1 replication compared to human leukocytes - Do not develop AIDS-like symptoms or immune suppression - Mice naturally control infection, including in CD4 + T cells	[150, 151]
Hu-PBL-SCID mice	Human peripheral blood lymphocytes (PBLs) injected i.p. into SCID mice	- HIV-1 can be injected i.p. (in PBLs or cell-free) - Easy technique and easy to gather human PBLs - HIV-1-mediated depletion of human CD4 + T cells, virus detectable in tissues that have been infiltrated by human cells -Clinically relevant anti-retroviral drug regimens can be directly tested - Study of latency reversal is feasible	- High percentage of GvHD (short life span) - All non-hematologic tissues are murine - No mucosal transmission of HIV-1 -Transient human B cells, myeloid cells and NK cells	[152, 153]
SRC-Hu mice	Human fetal thymus and liver cells transplanted into SCID mice, resulting in the production of human hematopoietic (CD34 +) progenitor stem cells	- HIV-1 can be injected into the human implants -Multilineage human immune reconstitution - HIV1-mediated depletion of human CD4 + T cells, virus detectable in tissues virus detectable and persistent in reservoirs -Relatively long lifespan and low percentage of GvHD - Clinically relevant anti-retroviral drug regimens can be directly tested - Study of latency reversal is feasible	- All non-hematologic tissues are murine - No mucosal transmission of HIV-1 - Leakiness of the model can lead to the generation of murine immune cells - Engraftment technique is moderately elaborated - Presence of innate immunity can cause engraftment rejection	[154–156]
BLT mice	NSG mice implanted with human fetal thymus and liver cells followed by sublethal irradiation and bone marrow transplantation with human CD34 + cells from the same donor	-Multilineage human immune reconstitution - HIV1-mediated depletion of human CD4 + T cells, virus detectable and persistent in reservoirs - Study of latency reversal is feasible - T cells develop within a human thymus, generating HLA-matched responses - HIV-1 infection can happen via mucosal tissue injection or directly in human implants - Clinically relevant anti-retroviral drug regimens can be tested	- All non-hematologic tissues are murine - Engraftment technique is elaborated, and the model is expensive	[157, 158]
EcoHIV	chimeric virus, recombinant version of HIV-1/NL4-3 where gp120 is replaced with gp80 (ecotropic murine leukemia virus)	-Virus is tropic for mouse macrophages, MDSCs, and T cells - Infected mice show persistent integration, infection, and immune suppression -Up-regulation of PD-1 and TIM-3 on CD8 + T cells, expansion of MDSCs in the spleen, and T_REG_ cells in the lymph nodes - HIV-associated neurocognitive impairments are detected - Safe, inexpensive, easy to manipulate -Physiological murine immune system	- Murine tissues and physiology - Infection is naturally controlled by mice and never evolves into AIDS-like illness (CD4^+^T cell/CD8^+^T cell ratios are unchanged)	[159–161]

While, theoretically, these models are amenable to use in cancer co-morbidity studies, crossing existing mouse models of HIV to GEMM cancer models is complex due to the potential involvement of many transgenes and mouse strain-specific compatibility of syngeneic tumor grafts.

Immunocompromised mouse models show appeal to study HIV and cancer co-morbidity since they allow for the engraftment of human cells into the mouse system without immune-mediated rejection. Humanized mice can be established, among others, using peripheral blood (Hu-PBL-SCID), human hematopoietic stem progenitor cells (SRC-Hu), or via tissue fragment transplantations of human fetal liver and thymus into NOD/SCID/IL2rγnull (NSG) mice followed intravenous transplantation of human fetal liver-derived CD34^+^hematopoietic stem cells (BLT model) [162]. The BLT model is the most advanced and elaborate, as the transplant process not only engrafts to the bone marrow, creating human lymphoid and myeloid cell compartments but also allows for human T cell education and immune reconstitution in mice. Some further genetically modified humanized mouse strains can even generate class-switched antigen-specific antibodies (humanized DRAGA model) [163]. Various humanized models have different levels of technical challenge, cost, and reproduction of the human immune system, but they are among the closest models to human disease. HIV infection is robust in these animals and can be treated with human-grade ART or antibodies. Most of the advanced models have CD4 + T cells and CD8 + T cells and macrophages infected by HIV-1, showing the classic development of human pathology (e.g., CD4 + T cell depletion). For example, the BLT model permits the formation of HIV latency following HIV infection and maintains persistent reservoirs of the virus, including latently infected CD4 + T cells after treatment with clinically relevant ART regimens [164]. BLT models can also be infected with HIV-1 using the most frequent way of transmission, such as intrarectal, intravaginal, and intravenous, but an alternative is to perform patient-derived xenograft models from HIV + donors, for example, utilizing an enriched population of CD4 + T memory cells [165].

The utilization of humanized models for HIV and cancer co-morbidity is theoretically feasible but retains several limitations. First, they have the issues of all humanized mouse models (e.g., limited lifespan, sample volumes, GvHD). In addition, most of the tissues outside the immune system are murine, and pharmacodynamics and pharmacokinetics differ from humans. There are also critical ethical limitations (e.g., BLT model), elevated costs, availability of hematopoietic stem cells, and challenging techniques, limiting the scalability. In addition, most of the humanized models need to be HLA-matched to avoid tissue/cancer cell rejection.

Several groups have attempted to produce transgenic mice expressing human CD4 and CCR5 to enable HIV-1 infection of murine cells. While HIV-1 infection occurs, it is controlled by the mice within a few weeks, and mice do not develop immune suppression [150, 151]. An alternative to modifying mice for HIV infection is to alter the virus itself. EcoHIV is a chimeric virus, a recombinant version of HIV-1/NL4-3 where the coding region of HIV envelope protein (gp120) is replaced with the envelope from ecotropic murine leukemia virus (gp80), a retrovirus that infects only rodents. EcoHIV is tropic for mouse macrophages, MDSCs, and T cells [159, 160]. EcoHIV establishes persistent infection in mice but does not cause AIDS as described in humans, as these animals maintain CD4 + T cell/CD8 + T cell ratios even after several months post-infection. EcoHIV-infected mice naturally control the infection and keep functional responses to HIV Gag peptides throughout infection. EcoHIV-infected animals retain resting CD4 + T cells in chronically infected mice as reservoirs for latent EcoHIV provirus. Notably, EcoHIV-infected mice show persistent integration, infection, and immune suppression [160], associated with up-regulation of PD-1 and TIM-3 on CD8 + T cells, expansion of MDSCs in the spleen, and T_REG_ cells in the lymph nodes. EcoHIV infection also causes HIV-associated neurocognitive impairments (HIV-NCI) and has been successfully used to evaluate this pathology [166, 167].

The EcoHIV model is suitable for HIV and cancer co-morbidity studies, utilizing allografts or GEMM to induce tumors after the viral infection. The advantage of this system is the utilization of fully immune-competent mice, allowing the evaluation of cancer and HIV interaction in a physiological immune system while improving cost-containment and scalability. The disadvantages are that the system does not fully recapitulate human disease (e.g., CD4 + T loss, mouse tumor cells), and it cannot be used to study AIDS-associated tumors, as it models a population of patients with stable provirus integration, a controlled infection, and normalized CD4 + T cell counts (e.g., PLWH on ART).

Overall, mouse models offer intriguing possibilities for HIV research even if they fall short in replicating the complete spectrum of HIV infection and AIDS progression seen in humans. As we described, each model possesses distinct advantages and limitations, but it is pivotal that we begin interrogating such animal systems to answer key biological questions in the field. Even with all their pitfalls, these models serve as invaluable surrogates, enabling researchers to simulate, manipulate, and observe biological processes in controlled environments that would be ethically and practically unattainable otherwise.

## Conclusions and future perspectives

**Fig. 3:**
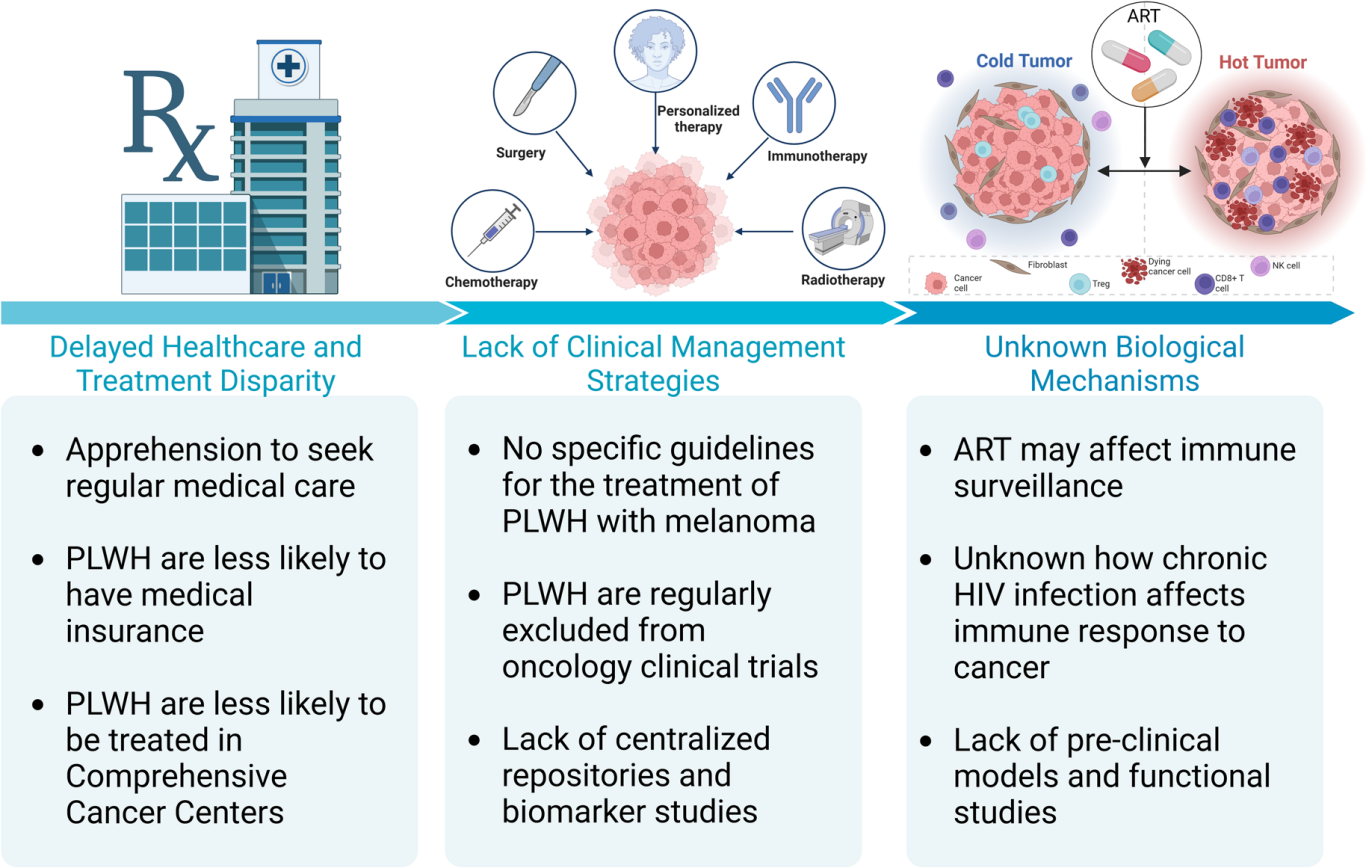
Main critical points that need to be addressed by clinical and pre-clinical research to address the issue of the increased mortality rate of PLWH with melanoma. PLWH, people living with HIV; ART, anti-retroviral therapy

The intricate interplay between HIV chronic infection and the immune landscape has profound implications for melanoma biology and clinical outcomes. The enduring legacy of HIV within the immune system, characterized by dysregulated functionalities and sustained inflammatory mediators, creates an environment conducive to immunological perturbations. This chronic impact extends beyond viral presence, fostering a milieu primed for an impaired immune response and the formation of an immunologically "cold" tumor microenvironment. Such complexities underscore the need for comprehensive research unraveling the biological underpinnings. Novel insights into immune dynamics, therapeutic influences, and the evolving landscape of cancer immunotherapy are central to fostering the improvement of the clinical management of PLWH with melanoma and other tumors. To improve the quality of care for PLWH and cancer, we need to step up both in pre-clinical and clinical research (Fig. 3). First, the field needs to utilize and improve current animal models of co-morbidity, as they are a crucial tool in deciphering biological intricacies and addressing clinical challenges. The utility of *in vivo* models in simulating and observing biological processes offers invaluable insights toward optimizing outcomes for the understudied yet critically important population of PLWH with melanoma. On the clinical side, it appears evident that immunotherapy is not less safe for PLWH compared to the general population, and recent studies in lung cancer have also preliminarily indicated similar efficacy [123]. Nevertheless, more participation in clinical trials is needed, as well as a centralized publicly available database of HIV and cancer, including clinically annotated patient samples and omics datasets. Such effort can only be attained through collaboration among academic institutions and hospitals, but it fundamentally requires the active involvement of the patients in the decision-making processes and experimental procedures. It is the role of institutions on the territory to work on the psycho-social factors limiting the participation of PLWH in research and to develop strong initiatives that put the patients first and use biomedical research as a tool to improve and prolong their lives.

## Data Availability

No datasets were generated or analysed during the current study.
